# Books Reviewed

**Published:** 1867-02

**Authors:** 


### Books Reviewed.

### Flint's Practice of Medicine.

We did not expect to be called upon so soon to repeat our expressions of approval of this work; a few months only and we receive a second edition. We observe by preface to second edition, "that notwithstanding the brief period allowed for a revision, additions have been made which, it is believed, will enhance the practical utility of the volume. The portion treating of Pyæmia has been re-written; three affections, omitted in the first edition, have been introduced, viz: Pertussis, General Cerebral Paralysis, and Polyuria; Epidemic Cholera has been considered at greater length; the thermometric phenomena of disease have received fuller consideration, and, in connection with many affections, there has been added new matter, much of which relates to special therapeutics." During the brief period which has elapsed since the publication of this work the profession have generally become more or less familiar with its teachings, and have shown their appreciation of its value. There was a long period of repose in authorship upon practical medicine; a time of so great change and expectation, that physicians stood, as it were, motionless, not venturing to go forward, lest they go wrong, not quite daring to stand still, because everything was moving, unwilling to turn backwards since revolutions were believed to never go that way. This was the "middle age" between, active aggressive warfare on disease, on the one hand, and prudent, rational care of mankind, when sick, on the other. The idea that disease is self-limited, and can often terminate favorably from inherent tendencies in this direction, provided its victims receive no greater privations and abuses than most well people are made to suffer, is still so new, that many physicians even, have not yet obtained a distinct understanding of it, and as a consequence remain in ignorance of facts, which it were much better to know. All diseases require to be understood, not so much that they may be treated with medicines, as that they may not be interfered with, by unnecessary annoyances; all sick people require care, and there is scarcely a condition of disease but some of its symptoms may be modified and its pains lessened by enlightened and judicious medication. It is to be hoped that the extreme of activity in medical practice which formerly prevailed will not be succeeded by another equally great extreme in the opposite direction.

Dr. Flint, who has been known in this country for many years, both as an author and teacher, who has discovered truth, and pointed it out clearly and distinctly to others, investigated the symptoms and natural history of disease and recorded its language and facts, and devoted a life of incessant study and thought to the doubtful or obscure in his profession, has at length, in his ripe scholarship, given this work to the profession as a crowning gift. If we have spoken highly of its value to the profession and world; if we have said, all considered, it is the very best work upon medical practice in any language; if we have spoken of its excellencies in detail, and given points of special value, we have yet failed to express in any degree our present estimate of its value as a guide in the practice of medicine. It does not contain too much or too little; it is not positive where doubt should be expressed, or hesitate where truth is known. It is philosophical and speculative where philosophy and speculation are all that can at present be obtained, but nothing is admitted to the elevation of established truth, without the most thorough investigation. It is truly remarkable with what even hand, this work has been written, and how it all shows the most careful thought and untiring study.

It will be asked, has this work in your estimation no faults? Yes, it probably has some faults, but what we might regard as its imperfections, and expect to see corrected in the next edition, many will regard otherwise, and hope to have remain unchanged. All cannot see at the same distance; the light is focused either in front of, or behind the retina. Our range of vision is not the standard measure, and it would be presumption for us to say, that our author sometimes tolerated measures of treatment which he could not recommend, since many others would think, he should recommend, what we are sorry to see even tolerated; so we conclude, that, though it may yet be susceptible of improvement, it still constitutes the very best which human knowledge can at present produce. "When knowledge is increased," the work will doubtless be again revised and enlarged; mean while we shall accept it as the rule of practice.

---

Practical Therapeutics. By Edward John Waring, F. R. C. S., F. L. S. Philadelphia: Lindsay & Blakiston, 1867,

The author says, "since the publication of the first edition of this work, the treatment of inflammatory and febrile affections has been in a transition state, diffusible stimulants having, in a great measure, re-placed blood-letting and other antiphlogistic remedies formerly in vogue. Although I have been unable fully to recognize the great asserted superiority of the new mode of treatment, to the total exclusion of other measures, which centuries of experience have proved to exercise valuable remedial powers, yet my own more extended experience, as well as the recorded cases of others, has conclusively shown that the old mode of treatment was capable of great improvement, and that we may have recourse, with manifest advantage, to stimulants at an earlier period, and in larger quantities than was formerly regarded either advisable or safe. A similar remark applies, with equal force, to the employment of quinine in the treatment of paroxysmal fevers, in which depletion and calomel are, in a great measure, replaced by the preparations of cinchona. These remarks are necessary to explain the modifications which some of the articles, as they appeared in the first edition, have undergone. It has been my earnest endeavor to hold the balance evenly—a work of no small difficulty, under the circumstances."

The above quotation gives our readers a better insight into the design and scope of this work than any other description, and we have given it in lehgth, becaiise so much is to be understood by the very little which is said.

This edition contains all the preparations of the new British Pharmacopia, together with notices of the principal new remedies which have been introduced into practice.

The design and scope of this work is thoroughly practical, and in fullness of therapeutical discussion is unsurpassed. It contains what is knowu of the therapeutical uses of all medicines, their sources, chemical and medicinal properties- doses, etc., etc. It is a very encouraging omen to receive works of this description; it shows that time corrects abuses and rectifies errors, that we are making progress in rational medicine.

---

Surgical Clinic of La Charité—Lessons upon the Diagnosis and Treatment of Surgical Diseases, delivered in the month of August, 1865, by Prof. VELPEAU, Membre de l' Institute at de l' Académie Impériale de Médicine. Collected and edited by A. REGNARD, Inteme des Hôspitaux. Reviewed by the Professor. Translated by W. C. B, Fifield, M D. Boston; James Campbell, 18 Tremont street; 1866.

The name of VELPEAU alone, recommends this book to the profession, and speaks more for it, than could any notice of ours.

The principal points treated of in this neat little work, are—1; Generalities. 2; Fractures. 3; Affections of the Joints. 4; Inflammation and Abcesses. 5; Affections of the Symphatic system. 6; Burns and Contusions. 7; Affections of the Geuito-urinary Organs. 8; Affections of the Anal Regions. 9; Affection of the Eyes. 10; Statistics of Operations. By the above synopsis, its value and usefulness will be readily seen and appreciated.

"It contains matter, which is useful for the medical Jurist, and the practitioner haunted by the fear of prosecution for mal-practice."

"To the student just embarking in his profession, this little monitor will tell what is done for Surgery, by the mighty force called nature, and how she is best aided by art."

Infantile Paralysis and its Attendant Deformities. By CHARLES FAYETTE TAYLOR, M. D. Philadelphia; J. B. Lippincott, & Co., 1867.

The author of this work appears to regard the various deformities so often arising in childhood, as paralysis, and this theory of the disease is carried over into the philosophy of a proposed cure. The more common deformities are represented by wood cut illustrations, and the various appliances are also represented, showing the chief means for rectifying deformities. The book is worthy of careful perusal, since it contains many useful suggestions, though it is not certain that all its conclusions and deductions are fully sustained by adequate evidence. Dr. Taylor has given great attention to the topics treated in this work, and his opinions are to be received with confidence. His modes of treatment are highly philosophical, and we have no doubt successful.

---

A treatise on the Principles and Practice of Medicine and Pathology, Diseases of Women and Children, and Medical Surgery. By W. PAYNE, M. D. Philadelphia.

Showing the character of this volume, we make the following quotation from the introductory. "New School physicians have discovered, and are using remedies which will enable them to cure a large per cent of cancer, hydrophobia, epilepsy, tetanus, scrofula, consumption, etc., all of which are regarded as incurable, under other systems of medicine, and justly styled by them the *approbrium medicorium*. Besides the New School cure all diseases, both acute and chronic, in a much less time, and with far more certainty than any other system." Further comments upon the character of this work are unnecessary; perhaps it is better for that denomination of physicians, than to have no guide at all.

---

The common nature of Epidemics, and their relation to Climate and Civilization. Also remarks on contagion and quarantine. From writings and official reports. By SOUTHWOOD SMITH, M. D., physician to London Fever Hospital, Consulting physician to the Hospital for Disease of the Skin, "the Father of Sanitary Reform," Member of the General Board of Health, 1848, 1854, &c., &c. Edited by T. Baker, Esq., of the Inner Temple, Barrister at Law, &c., &c. Philadelphia; J. P. Lippincott & Co., 1866.

The important subjects of Epidemics—Quarantine and Contagion—are treated in a masterly and scientific manner, and cannot fail to interest, as well s instruct all.

1. Taking the ground that epidemic diseases are universally and inseparably connected with an epidemic atmosphere, he claims that quarantine can exercise no more control over "this atmosphere" than over the electricity and temperature of the common air, and the direction of the wind.

2. Epidemics. Without accepting any of the numerous theories concerning epidemics, he considers them connected with the atmosphere, and that favoring causes, such as crowding, filth, &c., start and spread these diseases.

3. Contagion. Under this head, he gives many interesting statistics, with regard to the spread and extent of Contagious diseases both in this country and elsewhere.

